# Supplementary material for: From sequence to enzyme mechanism using multi-label machine learning
Source: BMC Bioinformatics. 2014 May 19;15:150. doi: 10.1186/1471-2105-15-150 (PMC4229970; doi:10.1186/1471-2105-15-150)
Supplement: Additional file 2 — Java code of ml2db. Additional file ml2db_code.tar.gz contains the Java source code to run the multi-label machine learning experiments and save the results to database. The code’s Javadoc is included. [file 1471-2105-15-150-S2.zip › additional file 2/ml2db/ml2db/doc/mulan/classifier/class-use/MultiLabelLearner.html]

Uses of Interface mulan.classifier.MultiLabelLearner


JavaScript is disabled on your browser.


- Overview
- Package
- Class
- Use
- Tree
- Deprecated
- Index
- Help

- Prev
- Next

- Frames
- No Frames

- All Classes

## Uses of Interface mulan.classifier.MultiLabelLearner

- Packages that use MultiLabelLearner

  | Package | Description |
  | --- | --- |
  |  |  |
  | --- | --- |
  | mulan.classifier |  |
  | mulan.classifier.lazy |  |
  | mulan.classifier.meta |  |
  | mulan.classifier.meta.thresholding |  |
  | mulan.classifier.neural |  |
  | mulan.classifier.transformation |  |
  | mulan.data |  |
  | mulan.evaluation |  |
- - ### Uses of MultiLabelLearner in mulan.classifier

    Classes in mulan.classifier that implement MultiLabelLearner

    | Modifier and Type | Class and Description |
    | --- | --- |
    |  |  |
    | --- | --- |
    | `class` | `MultiLabelLearnerBase` Common root base class for all multi-label learner types. |

    Methods in mulan.classifier that return MultiLabelLearner

    | Modifier and Type | Method and Description |
    | --- | --- |
    |  |  |
    | --- | --- |
    | `MultiLabelLearner` | MultiLabelLearnerBase.`makeCopy()` |
    | `MultiLabelLearner` | MultiLabelLearner.`makeCopy()` Creates a deep copy of the given learner using serialization. |
  - ### Uses of MultiLabelLearner in mulan.classifier.lazy

    Classes in mulan.classifier.lazy that implement MultiLabelLearner

    | Modifier and Type | Class and Description |
    | --- | --- |
    |  |  |
    | --- | --- |
    | `class` | `BRkNN` Simple BR implementation of the KNN algorithm.For more information, see |
    | `class` | `IBLR_ML` This class is an implementation of the "IBLR-ML" and "IBLR-ML+" methods for the MULAN package.    For more information, see    Weiwei Cheng, Eyke Hullermeier (2009). |
    | `class` | `MLkNN` Class implementing the ML-kNN (Multi-Label k Nearest Neighbours) algorithm.    For more information, see    Min-Ling Zhang, Zhi-Hua Zhou (2007). |
    | `class` | `MultiLabelKNN` Superclass of all KNN based multi-label algorithms |
  - ### Uses of MultiLabelLearner in mulan.classifier.meta

    Classes in mulan.classifier.meta that implement MultiLabelLearner

    | Modifier and Type | Class and Description |
    | --- | --- |
    |  |  |
    | --- | --- |
    | `class` | `ClusteringBased` Class implementing clustering-based multi-label classification. |
    | `class` | `EnsembleOfSubsetLearners` A class for gathering several different SubsetLearners into a composite ensemble model. |
    | `class` | `HMC` Class that implements a Hierarchical Multilabel classifier (HMC). |
    | `class` | `HMCNode` Class that implements a node for the `HMC` |
    | `class` | `HOMER` Class implementing the Hierarchy Of Multi-labEl leaRners algorithm. |
    | `class` | `MultiLabelMetaLearner` Base class for multi-label learners, which use other multi-label learners |
    | `class` | `RAkEL` Class implementing a generalized version of the RAkEL (RAndom k-labELsets) algorithm. |
    | `class` | `RAkELd` Class implementing a generalized version of the RAkEL-d (RAndom k-labELsets) algorithm with disjoint labelsets. |
    | `class` | `SubsetLearner` A class for learning a classifier according to disjoint label subsets: a multi-label learner (the Label Powerset by default) is applied to subsets with multiple labels and a single-label learner is applied to single label subsets. |

    Methods in mulan.classifier.meta that return MultiLabelLearner

    | Modifier and Type | Method and Description |
    | --- | --- |
    |  |  |
    | --- | --- |
    | `MultiLabelLearner` | MultiLabelMetaLearner.`getBaseLearner()` Returns the `MultiLabelLearner` which is used internally by the learner. |

    Constructors in mulan.classifier.meta with parameters of type MultiLabelLearner

    Constructor and Description

    `ClusteringBased(weka.clusterers.Clusterer aClusterer,
    MultiLabelLearner aMultiLabelClassifier)`

    Constructor

    `EnsembleOfSubsetLearners(MultiLabelLearner aMultiLabelLearner,
    weka.classifiers.Classifier aSingleLabelLearner,
    LabelPairsDependenceIdentifier aDependenceIdentifier,
    int models)`

    Initialize EnsembleOfSubset with multilabel and single label learners, a
    method for labels dependence identification and number of models to
    constitute the ensemble.

    `HMC(MultiLabelLearner baseLearner)`

    Constructs a new instance

    `HMCNode(java.lang.String name,
    MultiLabelLearner mlc)`

    Creates a new instance with the given name and learner

    `HOMER(MultiLabelLearner mll,
    int clusters,
    HierarchyBuilder.Method method)`

    Creates a new instance based on given multi-label learner, number of
    children and partitioning method

    `MultiLabelMetaLearner(MultiLabelLearner baseLearner)`

    Creates a new instance.

    `RAkEL(MultiLabelLearner baseLearner)`

    Creates an instance based on a given multi-label learner

    `RAkEL(MultiLabelLearner baseLearner,
    int models,
    int subset)`

    Creates an instance given a specific multi-label learner, number of
    models and size of subsets

    `RAkEL(MultiLabelLearner baseLearner,
    int models,
    int subset,
    double threshold)`

    Creates an instance given a specific multi-label learner, number of
    models, size of subsets and threshold

    `RAkELd(MultiLabelLearner baseLearner)`

    Construct a new instance based on the given multi-label learner

    `RAkELd(MultiLabelLearner baseLearner,
    int subset)`

    Constructs a new instance based on the given multi-label learner and
    size of subset

    `SubsetLearner(int[][] labelsSubsets,
    MultiLabelLearner multiLabelLearner,
    weka.classifiers.Classifier singleLabelClassifier)`

    Initialize the SubsetLearner with labels set partitioning, multilabel and
    single label learners.

    `SubsetLearner(LabelClustering clusteringMethod,
    MultiLabelLearner multiLabelLearner,
    weka.classifiers.Classifier singleLabelClassifier)`

    Initialize the SubsetLearner with a label clustering method, multilabel
    and single label learners.
  - ### Uses of MultiLabelLearner in mulan.classifier.meta.thresholding

    Classes in mulan.classifier.meta.thresholding that implement MultiLabelLearner

    | Modifier and Type | Class and Description |
    | --- | --- |
    |  |  |
    | --- | --- |
    | `class` | `ExampleBasedFMeasureOptimizer` This class takes the marginal probabilities estimated for each label by a multi-label learner and transforms them into a bipartition which is approximately optimal for example-based FMeasure. |
    | `class` | `Meta` Base class for instance-based prediction of a bipartition from the labels' scores |
    | `class` | `MetaLabeler` Class implementing the MetaLabeler algorithm. |
    | `class` | `MLPTO` Class that implements the Multi Label Probabilistic Threshold Optimizer (MLTPTO). |
    | `class` | `OneThreshold` Class that estimates a single threshold for all labels and examples. |
    | `class` | `RCut` Classs that implements RCut(Rank-based cut). |
    | `class` | `SCut` Class that implements the SCut method (Score-based local optimization). |
    | `class` | `ThresholdPrediction` Class that learns to predict a different threshold per exampleFor more information, see    Elisseeff, Andre, Weston, Jason: A kernel method for multi-labelled classification. |

    Constructors in mulan.classifier.meta.thresholding with parameters of type MultiLabelLearner

    Constructor and Description

    `ExampleBasedFMeasureOptimizer(MultiLabelLearner baseLearner)`

    The supplied multi-label learner should be able to output marginal
    probabilities.

    `Meta(MultiLabelLearner baseLearner,
    weka.classifiers.Classifier aClassifier,
    java.lang.String aMetaDatasetChoice)`

    Constructor that initializes the learner

    `MetaLabeler(MultiLabelLearner baseLearner,
    weka.classifiers.Classifier classifier,
    java.lang.String metaDataChoice,
    java.lang.String aClassChoice)`

    Constructor that initializes the learner

    `MLPTO(MultiLabelLearner baseLearner,
    ExampleBasedBipartitionMeasureBase EBBM)`

    `OneThreshold(MultiLabelLearner baseLearner,
    BipartitionMeasureBase aMeasure)`

    `OneThreshold(MultiLabelLearner baseLearner,
    BipartitionMeasureBase aMeasure,
    int someFolds)`

    `RCut(MultiLabelLearner baseLearner)`

    Creates a new instance of RCut

    `RCut(MultiLabelLearner baseLearner,
    BipartitionMeasureBase aMeasure)`

    Creates a new instance of RCut

    `RCut(MultiLabelLearner baseLearner,
    BipartitionMeasureBase aMeasure,
    int someFolds)`

    Creates a new instance of RCut

    `SCut(MultiLabelLearner baseLearner,
    BipartitionMeasureBase measure)`

    Creates a new instance of SCut

    `SCut(MultiLabelLearner baseLearner,
    BipartitionMeasureBase measure,
    int folds)`

    Constructor that initializes the learner with a base algorithm , Measure
    and num of folds

    `ThresholdPrediction(MultiLabelLearner baseLearner,
    weka.classifiers.Classifier classifier,
    java.lang.String metaDataChoice,
    int folds)`

    Constructor that initializes the learner
  - ### Uses of MultiLabelLearner in mulan.classifier.neural

    Classes in mulan.classifier.neural that implement MultiLabelLearner

    | Modifier and Type | Class and Description |
    | --- | --- |
    |  |  |
    | --- | --- |
    | `class` | `BPMLL` The implementation of Back-Propagation Multi-Label Learning (BPMLL) learner. |
    | `class` | `MMPLearner` Implementation of Multiclass Multilabel Perceptrons learner. |
  - ### Uses of MultiLabelLearner in mulan.classifier.transformation

    Classes in mulan.classifier.transformation that implement MultiLabelLearner

    | Modifier and Type | Class and Description |
    | --- | --- |
    |  |  |
    | --- | --- |
    | `class` | `AdaBoostMH` Implementation of the AdaBoost.MH algorithm based on Weka's AdaBoostM1. |
    | `class` | `BinaryRelevance` |
    | `class` | `CalibratedLabelRanking` Class implementing the Calibrated Label Ranking (CLR) algorithm. |
    | `class` | `ClassifierChain` Class implementing the Classifier Chain (CC) algorithm.    For more information, see    Read, Jesse, Pfahringer, Bernhard, Holmes, Geoff, Frank, Eibe: Classifier Chains for Multi-label Classification. |
    | `class` | `EnsembleOfClassifierChains` Class implementing the Ensemble of Classifier Chains(ECC) algorithm. |
    | `class` | `EnsembleOfPrunedSets` Class implementing the Ensemble of Pruned Sets algorithm(EPS) . |
    | `class` | `IncludeLabelsClassifier` A multilabel classifier based on the include labels transformation. |
    | `class` | `LabelPowerset` Class that implements a label powerset classifier |
    | `class` | `LabelsetPruning` Common functionality class for the PPT and PS algorithms |
    | `class` | `MultiClassLearner` |
    | `class` | `MultiLabelStacking` This class is an implementation of the (BR)^2 or Multi-Label stacking method.    For more information, see    Grigorios Tsoumakas, Anastasios Dimou, Eleftherios Spyromitros, Vasileios Mezaris, Ioannis Kompatsiaris, Ioannis Vlahavas: Correlation-Based Pruning of Stacked Binary Relevance Models for Multi-Label Learning. |
    | `class` | `PPT` Class implementing the Pruned Problem Transformation algorithm (PPT) . |
    | `class` | `PrunedSets` Class implementing the Pruned Sets algorithm (PS). |
    | `class` | `TransformationBasedMultiLabelLearner` |
  - ### Uses of MultiLabelLearner in mulan.data

    Methods in mulan.data that return MultiLabelLearner

    | Modifier and Type | Method and Description |
    | --- | --- |
    |  |  |
    | --- | --- |
    | `MultiLabelLearner` | GreedyLabelClustering.`getMultiLabelLearner()` |

    Constructors in mulan.data with parameters of type MultiLabelLearner

    Constructor and Description

    `GreedyLabelClustering(MultiLabelLearner aMultiLabelLearner,
    weka.classifiers.Classifier aSingleLabelLearner,
    LabelPairsDependenceIdentifier dependenceIdentifier)`

    Initialize the GreedyLabelClustering with multilabel and single label learners and a method
    for labels dependence identification.
  - ### Uses of MultiLabelLearner in mulan.evaluation

    Methods in mulan.evaluation with parameters of type MultiLabelLearner

    | Modifier and Type | Method and Description |
    | --- | --- |
    |  |  |
    | --- | --- |
    | `void` | Evaluator.`checkLearner(MultiLabelLearner learner)` |
    | `MultipleEvaluation` | Evaluator.`crossValidate(MultiLabelLearner learner, MultiLabelInstances data, int someFolds)` Evaluates a `MultiLabelLearner` via cross-validation on given data set with defined number of folds and seed. |
    | `MultipleEvaluation` | Evaluator.`crossValidate(MultiLabelLearner learner, MultiLabelInstances data, java.util.List<Measure> measures, int someFolds)` Evaluates a `MultiLabelLearner` via cross-validation on given data set using given evaluation measures with defined number of folds and seed. |
    | `Evaluation` | Evaluator.`evaluate(MultiLabelLearner learner, MultiLabelInstances data)` Evaluates a `MultiLabelLearner` on given test data set. |
    | `Evaluation` | Evaluator.`evaluate(MultiLabelLearner learner, MultiLabelInstances data, java.util.List<Measure> measures)` Evaluates a `MultiLabelLearner` on given test data set using specified evaluation measures |

- Overview
- Package
- Class
- Use
- Tree
- Deprecated
- Index
- Help

- Prev
- Next

- Frames
- No Frames

- All Classes
